# Immunotherapy landscape analyses of necroptosis characteristics for breast cancer patients

**DOI:** 10.1186/s12967-022-03535-z

**Published:** 2022-07-21

**Authors:** Honghao Yu, Wenchang Lv, Yufang Tan, Xiao He, Yiping Wu, Min Wu, Qi Zhang

**Affiliations:** Department of Plastic and Cosmetic Surgery, Tongji Hospital, Tongji Medical College, Huazhong University of Science and Technology, 1095 Jiefang Avenue, Wuhan, 430030 Hubei China

**Keywords:** Breast cancer, Necroptosis, Prognosis, Immunotherapy, Signature

## Abstract

Necroptosis plays a major role in breast cancer (BC) progression and metastasis. Besides, necroptosis also regulates inflammatory response and tumor microenvironment. Here, we aim to explore the predictive signature based on necroptosis-related genes (NRGs) for predicting the prognosis and response to therapies. Using Lasso multivariate cox analysis, we firstly established the NRG signature based on TCGA database. A total of 6 NRGs (FASLG, IPMK, FLT3, SLC39A7, HSP90AA1, and LEF1), which were associated with the prognosis of BC patients, were selected to establish our signature. Next, CIBERSORT algorithm was utilized to evaluate immune cell infiltration levels. We compare the response to immunotherapy using IMvigor 210 database, and also compared immune indicators in two risk groups via multiple methods. The biological function of IPMK was explored via in vitro verification. Finally, our results indicated that the signature was an independent prognostic indicator for BC patients with better efficiency than other reported signatures. The immune cell infiltration levels were higher, and the response to immunotherapy and chemotherapy was better in the low-risk groups. Besides, other immunotherapy-related factors, including TMB, TIDE, and expression of immune checkpoints were also increased in the low-risk group. Clinical sample validation showed that CD206 and IPMK in clinical samples were both up-regulated in the high-risk group. In vitro assay showed that IPMK promoted BC cell proliferation and migration, and also enhanced macrophage infiltration and M2 polarization. In summary, we successfully established the NRG signature, which could be used to evaluate BC prognosis and identify patients who will benefit from immunotherapy.

**Supplementary Information:**

The online version contains supplementary material available at 10.1186/s12967-022-03535-z.

## Introduction

Breast cancer (BC) remains the most common cancer in women around the world, and accounts for nearly 30% of female cancers [1]. According to the estimated data from Cancer statistics 2022, there will be 290,560 new cases and 43,780 deaths of BC in the United States this year [2]. Besides, BC also has high heterogeneity and a high rate of metastasis. Bone, lung, liver, and brain are the most common sites of BC metastasis [3]. The heterogeneity and metastasis of BC result in the difficulty of treatment. Existing BC classifications, including TNM and PAM50 classification, are difficult to predict the prognosis and treatment effect of patients [4].

Necroptosis is a type of programmed cell death usually manifested with morphological features of necrosis, which is mainly regulated by receptor-interacting serine-threonine kinase 3 (RIPK3) and mixed lineage kinase domain-like (MLKL) [5]. Necroptosis plays an important role in tumorigenesis, metastasis, and antitumor immunity [6]. Koo et al. demonstrated that the overexpression of RIPK3 induced by hypomethylating agents promoted necroptosis in BC and improved the effect of chemotherapy [7]. Parkin could promote the polyubiquitination of RIPK3, and parkin overexpression was associated with worse prognosis in BC patients [8]. Moreover, Park et al. also showed that the activation of necroptosis inhibited tripartite motif protein 28 (TRIM28) and then increased the production of immunostimulatory cytokine, which resulted in enhanced anti-tumor immunity [9]. These studies suggest that necroptosis-related genes (NRGs) are valuable predictors of prognosis and the efficacy of immunotherapy for BC patients, as well as promising treatment targets.

In recent studies, immunotherapy is proved to be a promising treatment for BC [10]. The FDA have approved immune checkpoint inhibitors (ICIs) against cytotoxic T-lymphocyte-associated antigen (CTLA-4), programmed cell death receptor 1 (PD-1), and programmed cell death 1 ligand 1 (PD-L1) for treatment in many types of cancers [11]. For BC therapy, immunotherapy elicits lasting effects in just a few patients and does not benefit the majority of patients [12]. Chen et al. demonstrated that pyroptosis-related molecules could be used to predict the bladder cancer response to ICIs. The association between NRGs and the BC response to ICIs has not been explored in previous studies. Therefore, it is intriguing to establish a reliable NRG signature to predict the progress of patients and their response to immunotherapy.

**Fig. 1:**
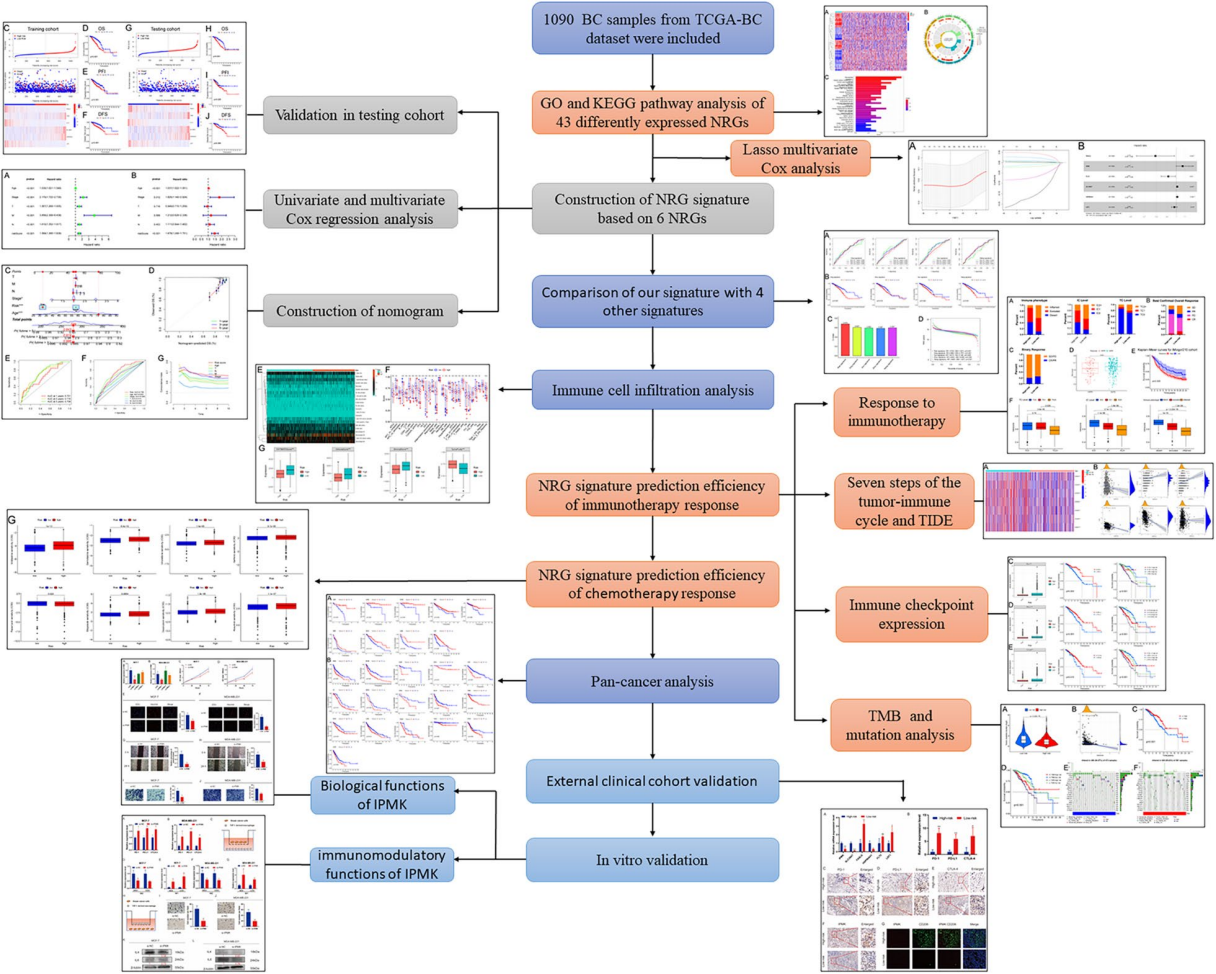
The flowchart of this study

Herein, we first established the NRG signature based on the Cancer Genome Atlas (TCGA) database. Subsequently, we tested its prediction efficiency for prognosis and response to immunotherapy and chemotherapy. In addition, an external clinical cohort including 20 BC patients was used to evaluate the signature. In vitro assay was performed to evaluate the role of selected gene inositol polyphosphate multikinase (IPMK) in BC progression and tumor immunity. The detailed flowchart was shown in Fig. 1. Our study is conducive to understanding the non-negligible role of necroptosis in regulating the BC tumor immune microenvironment, and provides a robust predictive method for those individuals that might benefit from specific optional BC immunotherapy.

## Materials and methods

### Data acquisition and preprocessing

The mRNA expression profiles and related clinical information of BC patients were extracted from TCGA database (https://cancergenome.nih.gov/). After excluding the patients without clinical information, 1090 BC patients in TCGA were finally enrolled in the analysis. 67 NRGs were extracted from gene card database. The GSE18728, GSE5462, and GSE20181 chip datasets and the related infusion-related reactions (IRRs) before and after chemotherapy were obtained from Gene Expression Omnibus (GEO). A database of urothelial cancer patients receiving anti-PD-L1 immunotherapy was obtained from the R package IMvigor 210 Core Biologies [13].

### Established and validation of NRG signature

To select the NRGs that were associated with the prognosis of BC patients, we firstly performed a univariate Cox regression analysis on the expression level of 67 NRGs and the overall survival (OS) of BC patients. Next, the LASSO regression was conducted to further compress the number of NRGs. Then, the NRG signature was established based on the Lasso multivariate Cox analysis. The formula of the NRG signature was as follows:

$$\mathrm{Risk score}=\sum_{i=1}^{n}{(\beta }_{i}*Expi)$$

where n was the number of NRGs, Exp indicated the NRG expression value of each BC patient and β was the regression coefficient of each NRG. According to the median risk score, BC patients were classified into low-risk and high-risk groups. The testing cohort, half of 1090 patients randomly selected from the training group, was utilized to verify the universality of our signature. In addition, we employed univariate and multivariate regression to identify the effects of risk scores and other clinical parameters on patient OS.

### The construction of a nomogram

Nomogram is a common tool used in the calculation of tumor prognosis, which shows the occurrence probability of individual clinical events by analyzing multiple prognostic and deterministic variables [14]. The nomogram that predicted 1-, 3-, and 5-year OS of BC patients was constructed based on the NRG signature and clinical parameters by the “rms” R package. In addition, we constructed calibration plots and area under the curve (AUC) to assess the accuracy of the nomogram in estimating patient prognosis.

### Immunologic infiltration analysis

To reveal the distinction of immune cell infiltration levels between the two risk groups, we calculated the fraction scores of 22 types of the immune cell using CIBERSORT algorithm [15]. Besides, the ESTIMATE scores, immune scores, stromal scores, and tumor purity were calculated by “estimate” R package [16].

### Immunotherapy response and chemosensitivity

The response to immunotherapy of patients in the two risk groups was obtained from IMvigor 210 database. After receiving immunotherapy, patients were classified into the following 4 types based on their response: stable disease (SD), partial response (PR), progressive disease (PD), and complete response (CR). To estimate the responses to chemotherapy in the two risk groups, we obtained the IC50 (Half of the maximum inhibitory concentration) values and the related mRNA profiles from the Genomics of Drugs Sensitivity in Cancer (GDSC). The chemosensitivity to commonly clinical chemotherapy drugs, including vinblastine, gemcitabine, vinorelbine, gefitinib, rapamycin, etoposide, doxorubicin, and bosutinib, was analyzed in our work.

### Mutation analysis

The “maftools” R package was used to calculate the tumor mutation burden (TMB) of BC patients. TMB was defined as the somatic mutation number per megabase of interrogated genomic sequence [17]. GISTIC2.0 was used to analyze the mutation and copy number alteration (CNA) of NRGs in BC samples.

### Quantitative real-time polymerase chain reaction (qRT-PCR)

The total RNA of BC tissues and cells were isolated by Trizol Reagent (Invitrogen, USA). The extracted RNA was then reverse-transcribed into complementary DNA (cDNA) with HiScript II 1st Strand cDNA Synthesis Kit (Vazyme Biotech, China). Next, we performed the qRT-PCR analysis with SYBR GreenTM Master Mix (Yeasen, China) and QuantStudio1 PCR (ABI Q1, USA) with a 10 μL system. All the primer sequences used for qRT-PCR were summarized in Additional file 1: Table S1.

### Western blot analysis

Total proteins from BC samples and cells were extracted with RIPA buffer (Beyotime, China). 12% sodium dodecyl sulfate–polyacrylamide gel electrophoresis (SDS-PAGE) was used to separate the total protein. The separated protein samples were then electrotransferred to polyvinylidene difluoride (PVDF) membranes (Bio-Rad, USA) and were blocked with 5% BSA for 1 h. Next, the PVDF membranes were incubated with primary antibodies at 4 °C overnight including anti-IL-4 (1:2000, Proteintech, USA), anti-IL-6 (1:2500 Proteintech, USA), and anti-β-tubulin (1:2500, Abcam, USA) antibodies. After washing the membranes 3 times with TBST, we incubated the membranes with HRP-conjugated secondary antibody (1:5000, Proteintech, USA) for 1 h. The enhanced chemiluminescence (ECL) assay kit (Yeasen, USA) was used to visualize the protein bands.

### Immunohistochemistry and immunofluorescence

After being fixed in the 10% formalin for 24 h, BC samples were sectioned at 4 μm. Immunohistochemistry (IHC) was performed with anti-IPMK (1:200, Proteintech, China), anti-PD-1 (1:100, Proteintech, USA), anti-PD-L1 (1:200, Proteintech, USA) and anti-CTLA-4 (1:100, Proteintech, USA) antibodies as primary antibodies according to the manufacturer’s protocols. For immunofluorescence (IF), anti-IPMK (1:200, Proteintech, USA) and anti-CD206 (1:100, Proteintech, USA) were used as primary antibodies to show the coexpression of CD206 and IPMK in the BC samples.

### Assay for proliferation and migration

The BC MCF-7 and MDA-MB-231 cells were transfected with 3 IPMK siRNAs (Ribobio Company, China) and negative control siRNA using Lipofectamine 3000 (Invitrogen, USA) to knock down the IPMK expression according to the manufacturer’s protocols.

The proliferation of MCF-7 and MDA-MB-231 cells were assessed with the cell counting kit-8 (CCK-8) assay (Dojindo, Japan). About 5 × 10^3^ cells were inoculated into 96-well plates, and cultured for 24, 48, and 72 h. Subsequently, 10 μL CCK-8 reagent was added to each well for 2 h, and the absorbance at 450 nm was used to measure the cell proliferation capacity.

The 5-Ethynyl-20-Deoxyuridine (EdU) incorporation assay kit (RiboBio, China) was also used to measure the proliferation capacity of cells. About 5 × 10^5^ cells were seeded into 12-well plates per well. After 24 h, the cells were incubated with culture medium containing EdU for 2 h and fixed with 4% paraformaldehyde for 30 min. After neutralizing the excess paraformaldehyde with 2 mg/mL glycine, we respectively incubated these cells in Apollo reaction cocktail and Hoechst staining solution for 30 min in the dark. A fluorescence microscope (Olympus, Japan) was utilized to capture images.

Wound healing assay and transwell migration assay were performed to measure the cell migration ability. For wound healing assay, 5 × 10^5^ cells were seeded in 12-well plates. When the cells grew to reach confluence, we scratched the cell layer with the tip of a 1 mL pipette and washed it with PBS 3 times. Scratch width change was measured under a 4 × microscope after 48 h of culture in serum-free DMEM medium. Transwell 24-wells plates (8 μm size, Corning, USA) were used for transwell migration assay. About 1 × 10^5^ cells with serum-free DMEM medium were uniformly seeded in the upper chamber, and 500 μL DMEM medium with 20% FBS was added into the basolateral chamber. After 24 h, the migrated cells were fixed with 4% paraformaldehyde for 30 min and then stained with 0.5% crystal violet for 30 min. Image J software (NIH, USA) was used to quantify the ability of cell migration.

### Macrophage generation and polarization

THP-1 cells were obtained from American Type Culture Collection (ATCC, USA). THP-1 cells which were induced with 50 ng/ml Phorbol-12-myristate-13-acetate (PMA) for 48 h were used as M0 macrophages. For the co-culture experiment, macrophages were seeded in the basolateral chamber of transwell 6-wells plates (0.4 μm size, Corning, USA), and BC cells were inoculated on the upper chamber. After 48 h, the cells were harvested for the follow-up experiments.

### Statistical analysis

The differences between the two groups were determined by Student’s t-tests and Wilcoxon Signed-rank test. The Chi-square test was performed to identify the correlations between the risk score and other clinicopathological parameters. The significance of differences between two Kaplan–Meier survival curves was determined by the log-rank test. All the statistical analyses were conducted by R software 3.6.3. The p < 0.05 (two-tailed) was considered significant significance.

## Results

### Enrichment analysis of differently expressed NRGs

BC cases from TCGA were used to construct the NRG signature. 43 differently expressed NRGs were shown in the heat map (Additional file 4: Figure S1A). The top 5 enriched terms of biological process (BP) of differently expressed NRGs were neuron death, programmed necrotic cell death, necrotic cell death, necroptotic process, and positive regulation of proteolysis. The top 5 molecular function (MF) terms were ubiquitin protein ligase binding, ubiquitin-like protein ligase binding, NAD binding, tumor necrosis factor receptor superfamily binding, and tumor necrosis factor-activated receptor activity. The top 5 cellular component (CC) terms were heterochromatin, CD40 receptor complex, membrane raft, membrane microdomain, and main axon (Additional file 4: Figure S1B). KEGG analysis showed that the pathways associated with tumor, virus infection, and apoptosis, such as necroptosis, human cytomegalovirus infection, and bladder cancer, were enriched in NRGs. Besides, TNF and NF-kB signaling pathways were enriched in differently expressed NRGs (Additional file 4: Figure S1C).

### Construction and validation of the NRG signature

**Fig. 2:**
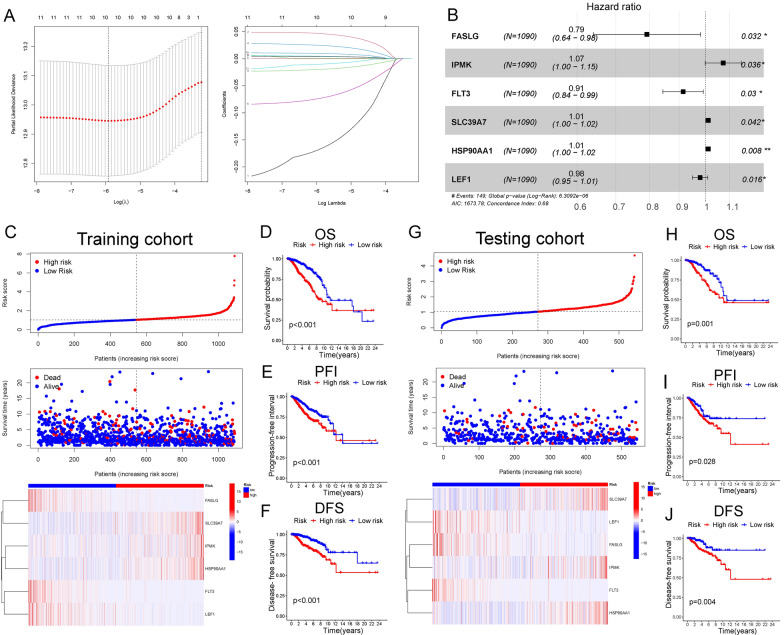
Construction and validation of the NRG signature. **A** LASSO Cox regression (with minimized lambda) of the differently expressed NRGs. **B** Forest plot showing the association between the expression level of 6 selected NRGs and the OS of patients. **C** Patient statuses and expression patterns for 6 selected NRGs in high- and low-risk groups of the training cohort. Kaplan–Meier (K-M) survival curve showing different OS (**D**), PFI (**E**), and DFS (**F**) in the two risk groups of the training cohort. **G** Patient statuses and expression patterns for 6 selected NRGs in high- and low-risk groups of the testing cohort. K-M survival curve showing different OS (**H**), PFI (**I**), and DFS (**J**) in the two risk groups of the testing cohort. *P < 0.05; **P < 0.01

Based on univariate Cox regression, we selected 43 differently expressed NRGs associated with the OS of patients. LASSO Cox regression (with minimized lambda) was performed to establish the NRG signature with 6 NRGs (FASLG, IPMK, FLT3, SLC39A7, HSP90AA1, and LEF1) (Fig. 2A, B, Additional file 2: Table S2, Additional file 3: Table S3). The formula of the signature was as follow:

$${\text{Risk}}\,{\text{score}} = {\text{IPMK}} * 0.07034 - {\text{FASLG}} * 0.23338 - {\text{FLT3}} * 0.08917 + {\text{SLC39A7}} * 0.00463 + {\text{HSP90AA1}} * 0.00088 - {\text{LEF1}} * 0.02173$$

The risk score of every BC patient was calculated with the formula. Then, BC patients were divided into high- and low-risk groups based on the median risk score (2.27). The survival time and the expression of selected NRGs were significantly different in the two risk groups (Fig. 2C). The high-risk group showed significantly worse OS, progression-free interval (PFI), and disease-free survival (DFS) (Fig. 2D–F). The testing cohort was used to validate the signature. The risk score of patients in the testing cohort was calculated with the same methods, indicating a relatively low OS, PFI, and DFS in the high-risk group (Fig. 2G–J). The risk signature was also with an excellent prediction for the prognosis of BC patients in the testing cohort.

### Validating the independence and universality of our signature

**Fig. 3:**
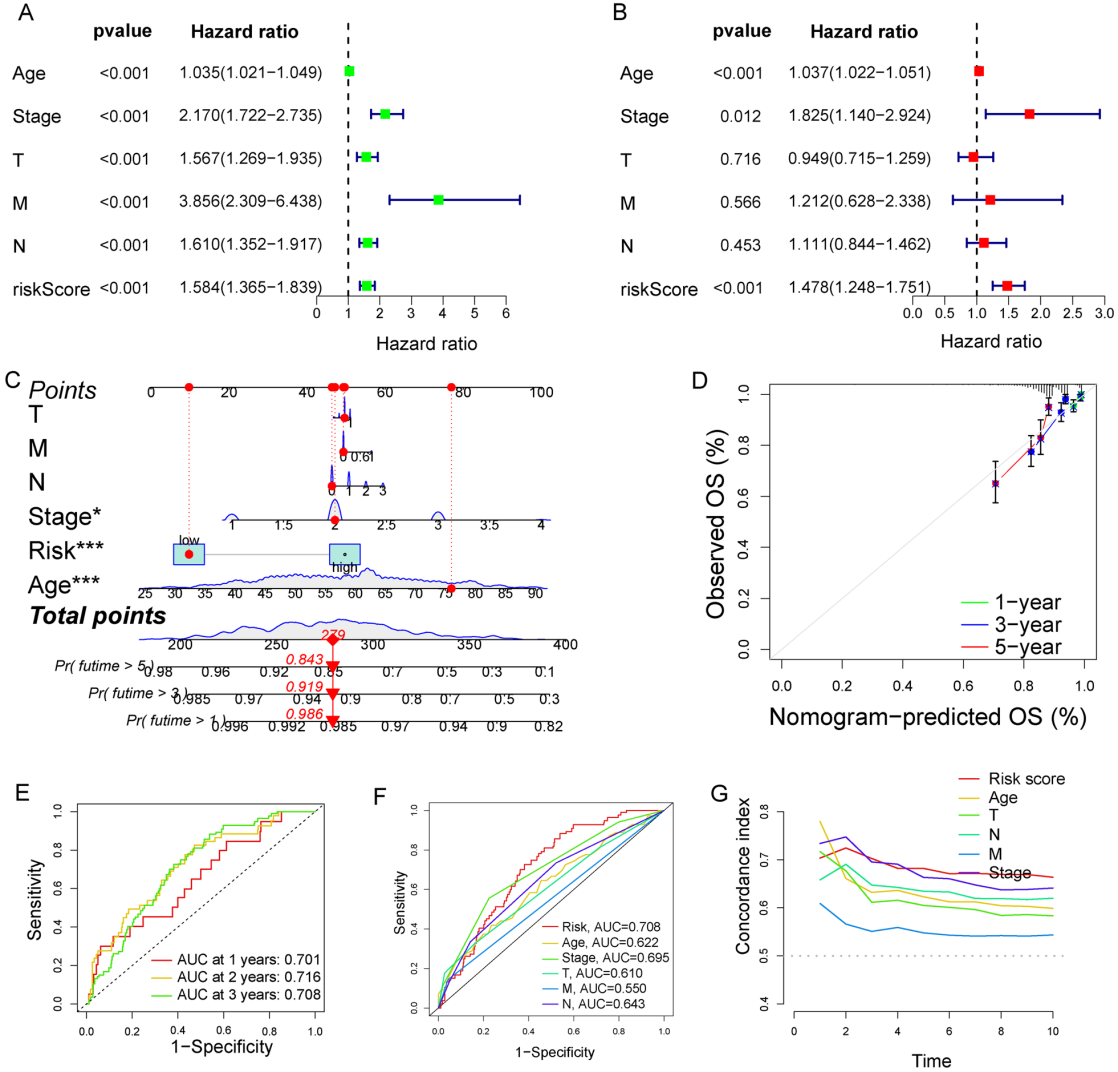
Construction of the nomogram. Univariate (**A**) and multivariate (**B**) Cox regression analysis for the risk score and other clinical characteristics. **C** Construction of the nomogram predicting the survival possibilities at 1-, 3-, and 5-years. **D** Correction curve showing the consistency between predicted survival possibilities and the observed survival rate. **E** ROC curve of our signature at 1-, 2-, and 3-years. **F** ROC curve of our risk score and other clinical characteristics at 3 years. **G** Comparison of the C-index of our risk score and other clinical characteristics

**Fig. 4:**
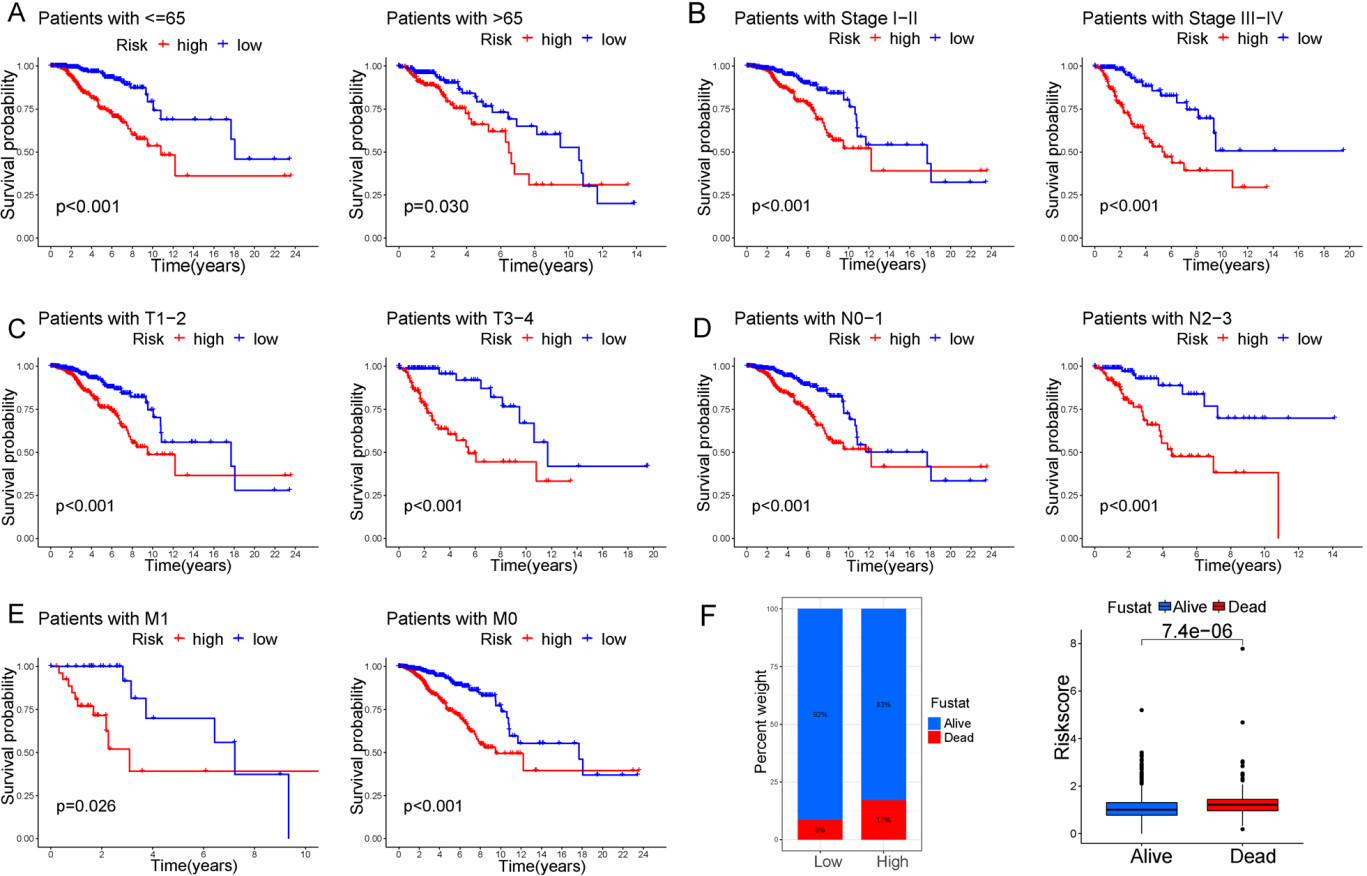
The prediction efficiency of the NRG signature in patients with different clinical states. **A** K-M survival curve of the high- and low-risk groups of different ages. **B** K-M survival curve of the two risk groups in different stages. **C** K-M survival curve of the two risk groups in different T categories stages. **D** K-M survival curve of the two risk groups in different N categories stages. **E** K-M survival curve of the two risk groups in different M categories stages. **F** Correlations between the risk scores and status

Using the univariate and multivariate Cox regression analysis, we proved that our risk score was an independent prognostic indicator for the OS of BC patients (Fig. 3A, B). The nomogram based on T, M, N, stage, risk score, and age was established to predict the survival rate at 1-, 3-, and 5-years of BC patients (Fig. 3C). The correction curve showed that the survival rate predicted by the graph was consistent with the observed survival rate (Fig. 3D). The AUC at 1-, 2-, and 3-years of NRG signature was 0.701, 0.716, and 0.708 (Fig. 3E). In comparison with other clinical characteristics, our signature was with the highest AUC and C-index (Fig. 3F, G). To verify the universality, the signature was performed on patients with different ages, stages, and TNM classifications. It found that our signature had a good prediction effect in every group of patients (Fig. 4A–E). Moreover, there were fewer deaths in the low-risk group (Fig. 4F).

### Comparison of the risk signature with others

**Fig. 5:**
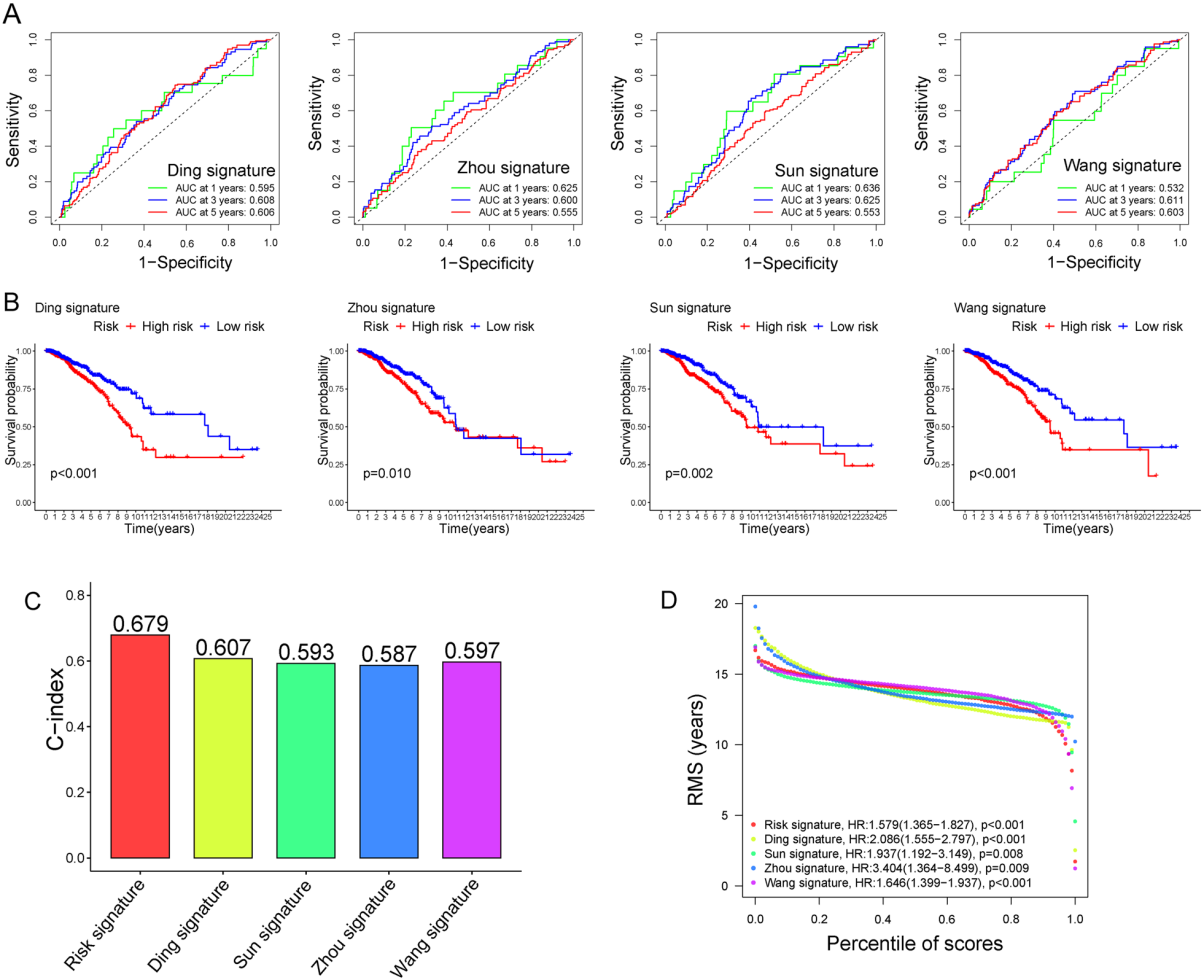
Comparison with other risk signatures. **A** ROC curve of Ding signature, Zhou signature, Sun signature, Wang signature. **B** K-M survival curve of the four signatures. **C** C-index of our risk signature compared with the four other signatures. **D** Restricted mean survival (RMS) curves for the five risk signatures

A total of 4 signatures in previous studies, Ding signature, Sun signature, Zhou signature, and Wang signature, were selected to compare with our signature [18–21]. In order to make these signatures comparable, the risk scores of each signature were calculated with the same methods. The results showed that our signature was with the highest AUC at 1-, 3-, and 5-years and C-index, and the prognostic difference between the two risk groups was more significant in our signature (Fig. 5A–C). The hazard ratio and p-value of the five signatures were presented in Fig. 5D. In summary, our model showed a better prognostic prediction performance in comparison to previous models.

### PAM50 subtypes and immune cell infiltration in high-risk and low-risk groups

**Fig. 6:**
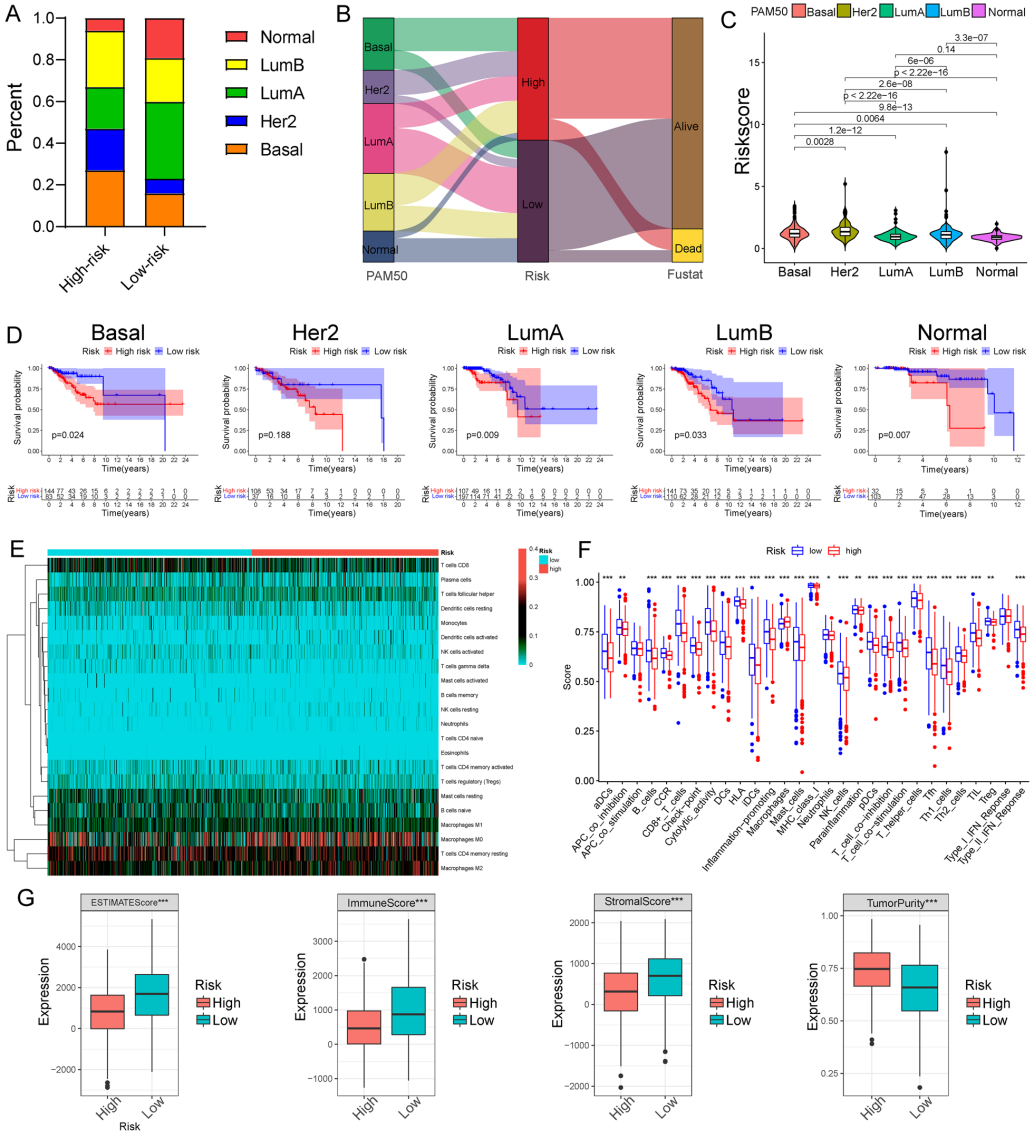
Immune cell infiltration and PAM50 subtypes in high-risk and low-risk groups. **A** Bar graphs for the distribution of PAM50 subtypes in the two risk groups. **B** Alluvial diagram for the different PAM50 subtypes and status in the two risk groups. **C** Violin plots for the risk scores of patients with different subtypes. **D** K-M survival curve of the five PAM50 subtypes. **E** Heat map for immune cell infiltration in the two risk groups. **F** The difference of immune cell infiltration scores in the two risk groups. **G** Boxplots showing ESTIMATE scores, immune scores, stromal scores, and tumor purity in the two risk groups. *P < 0.05; **P < 0.01; ***P < 0.001.

The proportion of every PAM50 subtype was significantly different between the two risk groups (Fig. 6A, B). The previous study had proved that Her2 and Basal types were related to a more aggressive phenotype, and worse prognosis, while BC patients with luminal A type always showed a better outcome [22]. Our results indicated that Her2 and Basal types were with higher risk scores compared with the luminal A type (Fig. 6C). The KM curve showed that our signature had prediction effects on prognosis in Basal, luminal B, luminal A, and normal types (Fig. 6D). Then, we calculated the fraction scores of 22 types of immune cells of each BC patient with CIBERSORT algorithm. Immune cell infiltration analysis indicated that the fraction scores of most immune cells, including B cells, NC cells, CD8 + T cells, and mast cells, were significantly higher in the low-risk group. However, macrophage infiltration was higher in the high-risk groups (Fig. 6E, F). In addition, the ESTIMATE scores, immune scores, and stromal scores were higher in the low-risk group, while tumor purity was higher in the high-risk group (Fig. 6G).

### Prediction of immunotherapy and chemotherapy treatment effects by the risk signature

**Fig. 7:**
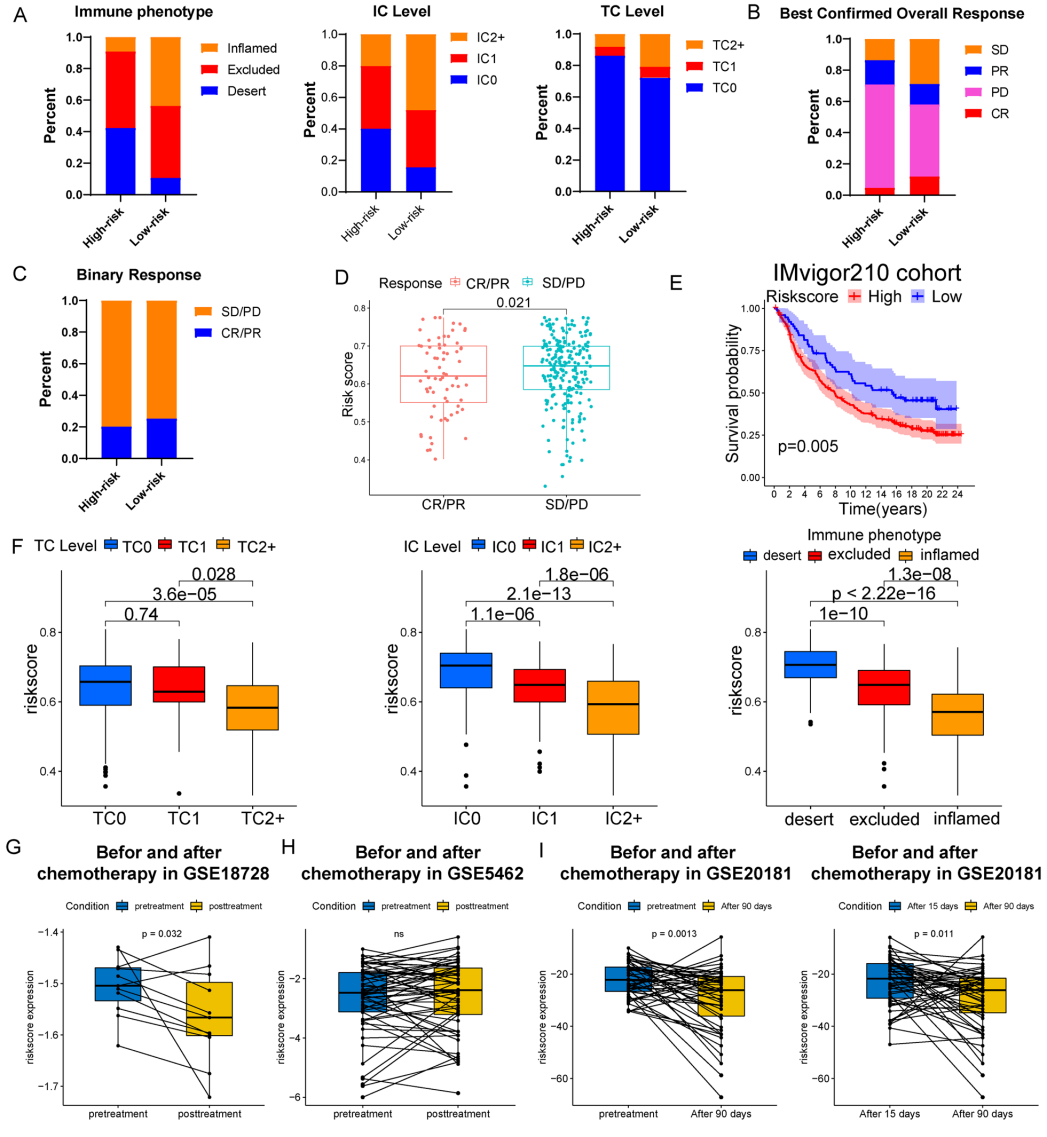
The responses to ICIs in high- and low-risk groups. **A** Bar graphs for the distribution of immune phenotype, IC level, and TC level in the two risk groups. **B**, **C** Bar graphs for the distribution of 4 types of response (SD, PR, PD, and CR). **D** Boxplots for the risk score in SD/PD and CR/PR groups. **E** K-M survival curve between the two risk groups in IMvigor 210 database. **F** Boxplots for the risk scores of patients with different immune phenotypes, IC level, and TC level. Boxplots for the paired risk score before and after chemotherapy in GSE18728 (**G**), GSE5462 (**H**), and GSE20181 (**I**) cohort

IMvigor 210 database was used to analyze immunotherapy response in the two risk groups. The distribution of immune phenotype, immune cells (IC) level, and tumor cells (TC) level in the two risk groups was presented in Fig. 7A. The patients with desert immunophenotype or lower IC and TC levels were concentrated in the high-risk group. In addition, the distribution of 4 immunotherapy response types (SD, PR, PD, and CR) was different in the two risk groups (Fig. 7B). The percentage of CR/PR was higher in the low-risk group and patients with CR/PR also had lower risk scores (Fig. 7C, D). The KM curve revealed a higher OS of the low-risk group in IMvigor 210 database (Fig. 7E). Also, patients with lower IC and TC levels and desert immunophenotype had the highest level of risk score (Fig. 7F). These data indicated that the low-risk group responded better to immunotherapy and was with lower IC and TC levels. The paired risk scores before and after neoadjuvant chemotherapy were acquired from GSE18728, GSE5462, and GSE20181 cohorts. The result showed that the risk score of the majority of patients was reduced after neoadjuvant chemotherapy (Fig. 7G–I).

**Fig. 8:**
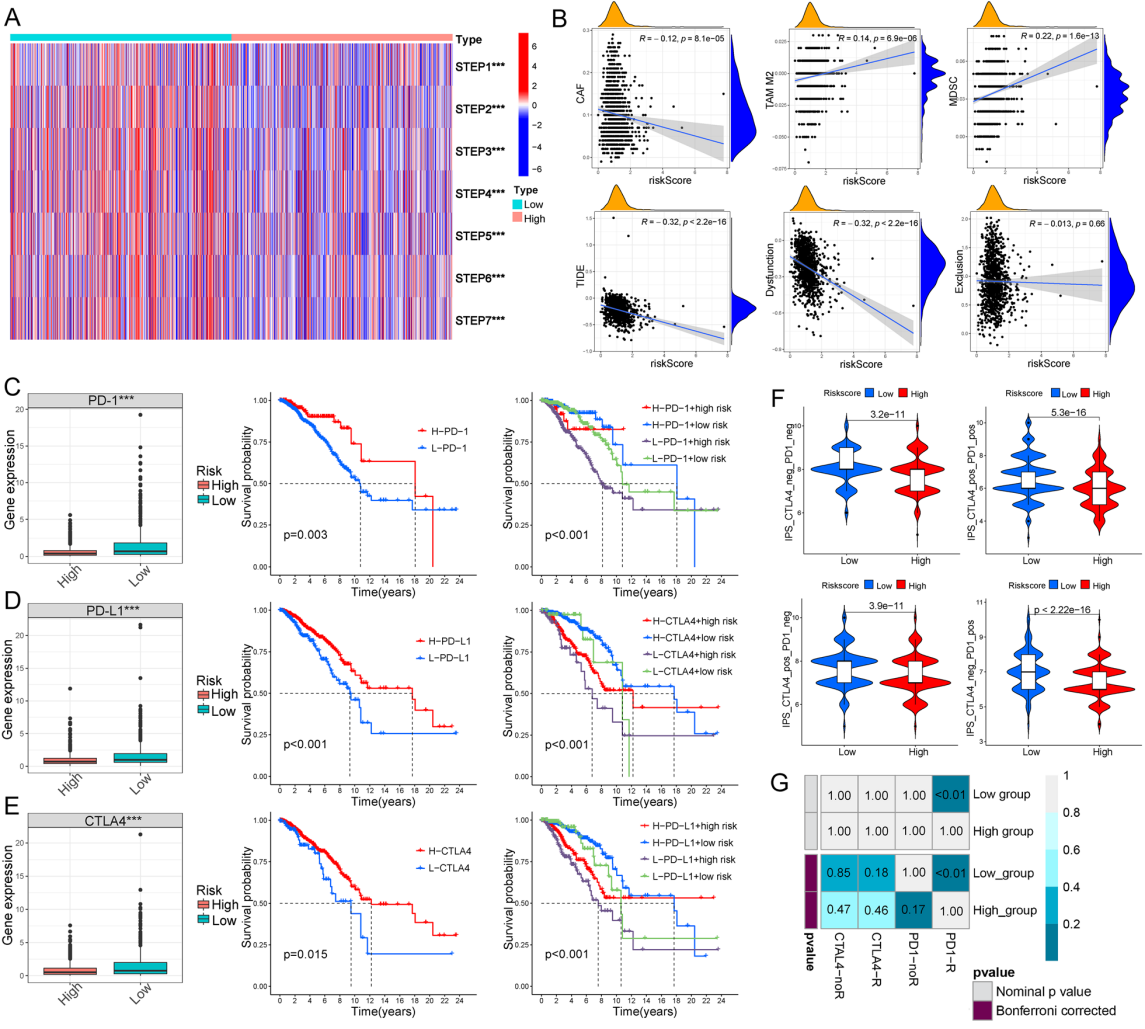
The NRG signature predicted the responses to ICIs. **A** Heat map for scores of seven tumor-immune cycle steps in high- and low-risk groups. **B** Correlations between the risk scores, CAF, TAM, MDSC, TIDE scores, dysfunction, and exclusion. **C** Correlations between the risk scores and the expression levels of PD-1 and the KM survival curve in groups divided by PD-1 expression and risk scores. **D** Correlations between the risk scores and the expression levels of PD-L1 and the KM survival curve in groups divided by PD-L1 expression and risk scores. **E** Correlations between the risk scores and the expression levels of CTLA-4 and the KM survival curve in groups divided by CTLA-4 expression and risk scores. **F** Violin plots for ips of 4 subtypes divided by the response to anti-PD-1 and anti-CTLA4 immunotherapy in the two risk groups. **G** Heat map for the response possibility of anti-PD-1 and anti-CTLA-4 treatment in the two risk groups. ***P < 0.001

The seven steps of the tumor-immune cycle were composed of [1] release of tumor antigens, [2] antigen presentation, [3] priming and activation, [4] trafficking of T cells, [5] infiltration of T cells, [6] cancer cells recognition by T cells, and [7] cancer cells killing [23]. The scores of the seven steps were all significantly higher in the low-risk group (Fig. 8A). In addition, cancer-associated fibroblast (CAF) level was negatively related to the risk score, tumor-associated macrophage (TAM), while myeloid-derived suppressor cells (MDSC) levels were positively related to the risk score. The risk score was also negatively correlated with TIDE scores, dysfunction, and exclusion (Fig. 8B). Previous studies demonstrated that the patients with higher TIDE scores were more likely to benefit from immunotherapy [24]. In addition, the expression levels of immune checkpoint PD-1, PD-L1, and CTLA-4 were higher in the low-risk group and positively related to the prognosis of BC patients (Fig. 8C–E). To predict the responses to ICI, we calculated the machine learning-based score (IPS) of 4 subtypes (CTLA-4_neg_PD-1_neg, CTLA-4_pos_PD-1_pos, CTLA-4_pos_PD-1_neg, and CTLA-4_neg_PD-1_pos). BC patients from the low-risk group were more likely to respond to anti-PD1, anti-CTLA-4, and combination treatment (Fig. 8F). Using the SubMAP algorithm, we confirmed that the low-risk group might respond better to anti-PD-1 treatment (Fig. 8G).

**Fig. 9:**
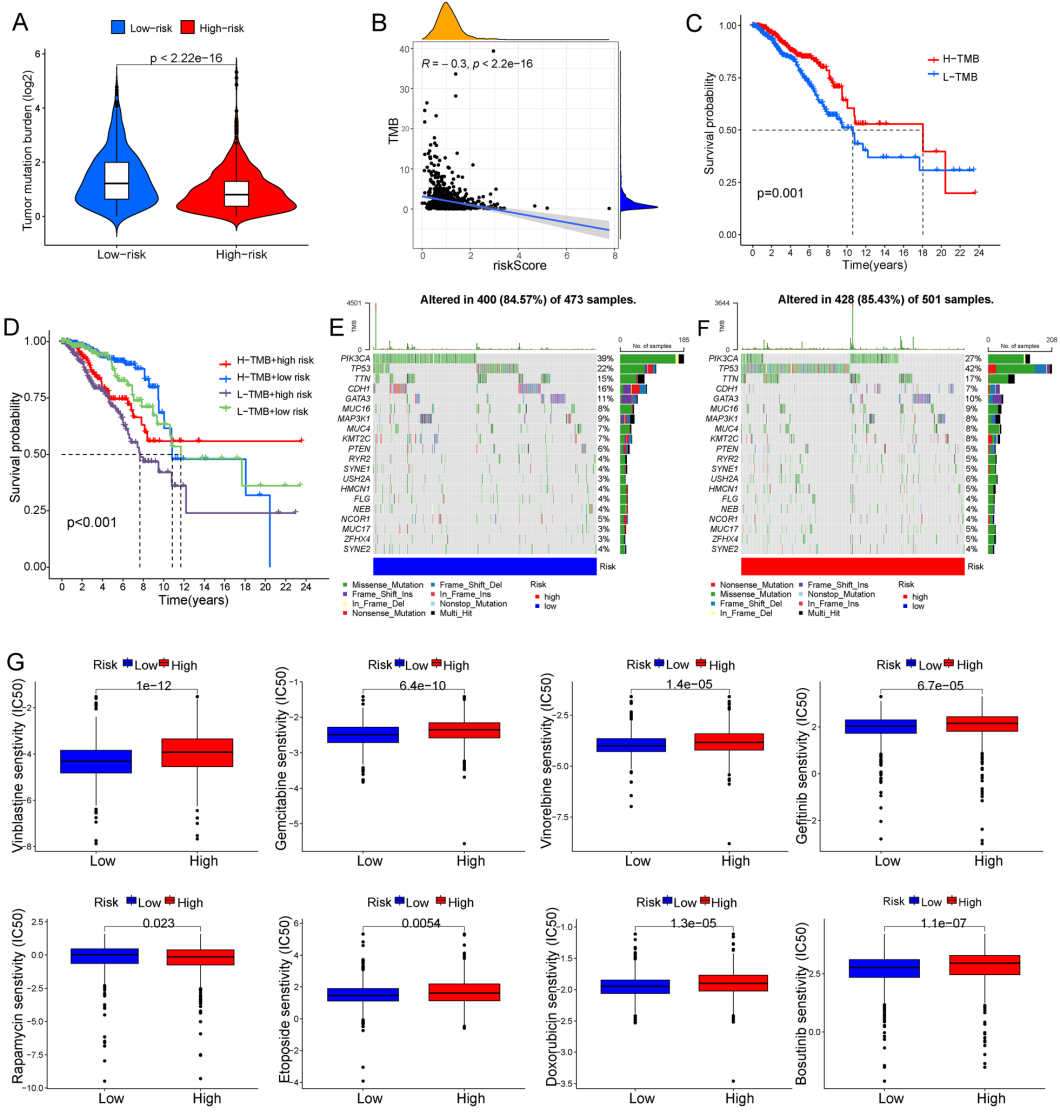
The NRG signature was associated with TMB and could predict the response to chemotherapy. **A** Violin plots for TMB between the two risk groups. **B** Correlations between risk scores and TMB. **C** KM survival curves in the high- and low- TMB groups. **D** KM survival curves among four groups divided by TMB and risk scores. Heat map visualization showed the top 20 frequently mutated genes in the high- (**E**) and low- (**F**) risk groups. **G** Boxplots for the response to vinblastine, gemcitabine, vinorelbine, gefitinib, rapamycin, etoposide, doxorubicin, and bosutinib in two risk groups

TMB is an important predictor of immunotherapy and chemotherapy. Patients with higher TMB will produce more neoantigens which might be potential targets for immunotherapy and chemotherapy [25]. We found that TMB was negatively related to the risk score and positively related to the prognosis of BC patients (Fig. 9A–D). Moreover, the top 20 frequently mutated genes in the two risk groups were presented in the waterfall chart (Fig. 9E, F). The mutation frequencies of these genes were significantly different between the two groups. IC50 values were calculated to predict the response to chemotherapy treatment. The results revealed that the low-risk group response better to vinblastine, gemcitabine, vinorelbine, gefitinib, etoposide, doxorubicin, and bosutinib. But the response to rapamycin was better in the high-risk group (Fig. 9G).

### Extending the risk signature from BC to Pan-Cancer

**Fig. 10:**
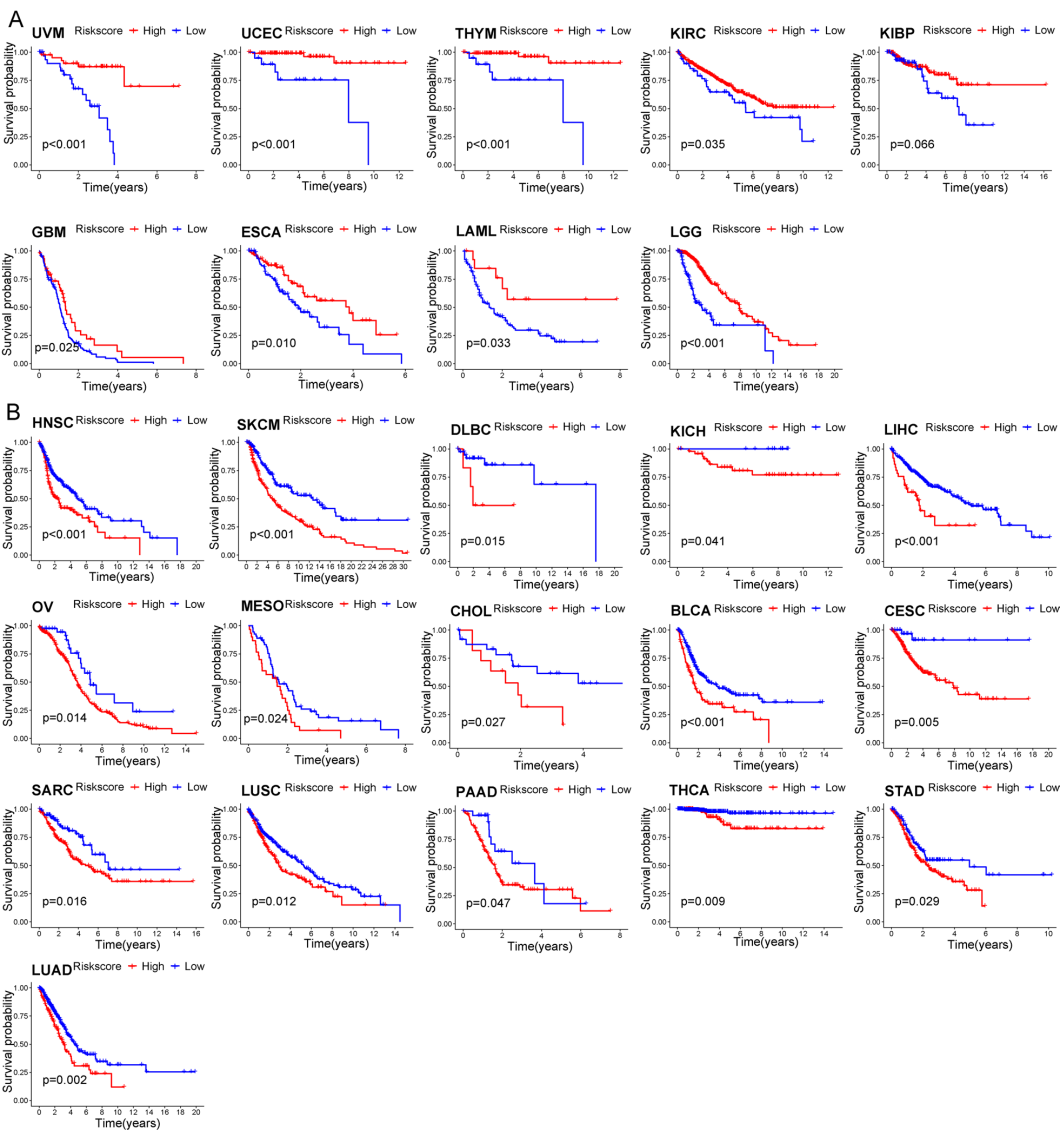
Prediction efficiency of the NRG signature in other 25 types of cancers. The KM survival curve analysis was performed to compare the OS of patients in the high-and low-risk group. The risk score was positively related to the OS in 9 types of cancers (**A**) and was negatively related to the OS in 16 types of cancers (**B**)

To explore the universality of the risk signature in other cancers, we used the above risk signature to calculate the risk score of patients suffering from other 32 cancer types in TCGA. The KM curve analysis showed that among the 32 types of cancer, the OS of patients in 25 cancer types was significantly related to the NRG signature. The high-risk group had a better prognosis in UVM, UCEC, THYM, KIRC, KIBP, GBM, ESCA, LAML, and LGG, while the patients in the high-risk group were with worse prognosis in HNSC, SKCM, DLBC, KICH, LIHC, OV, MESO, CHOL, BLCA, CESC, SARC, LUSC, PAAD, THCA, STAD, and LUAD (Fig. 10A, B).

### Validation of the risk signature in an external clinical cohort

**Fig. 11:**
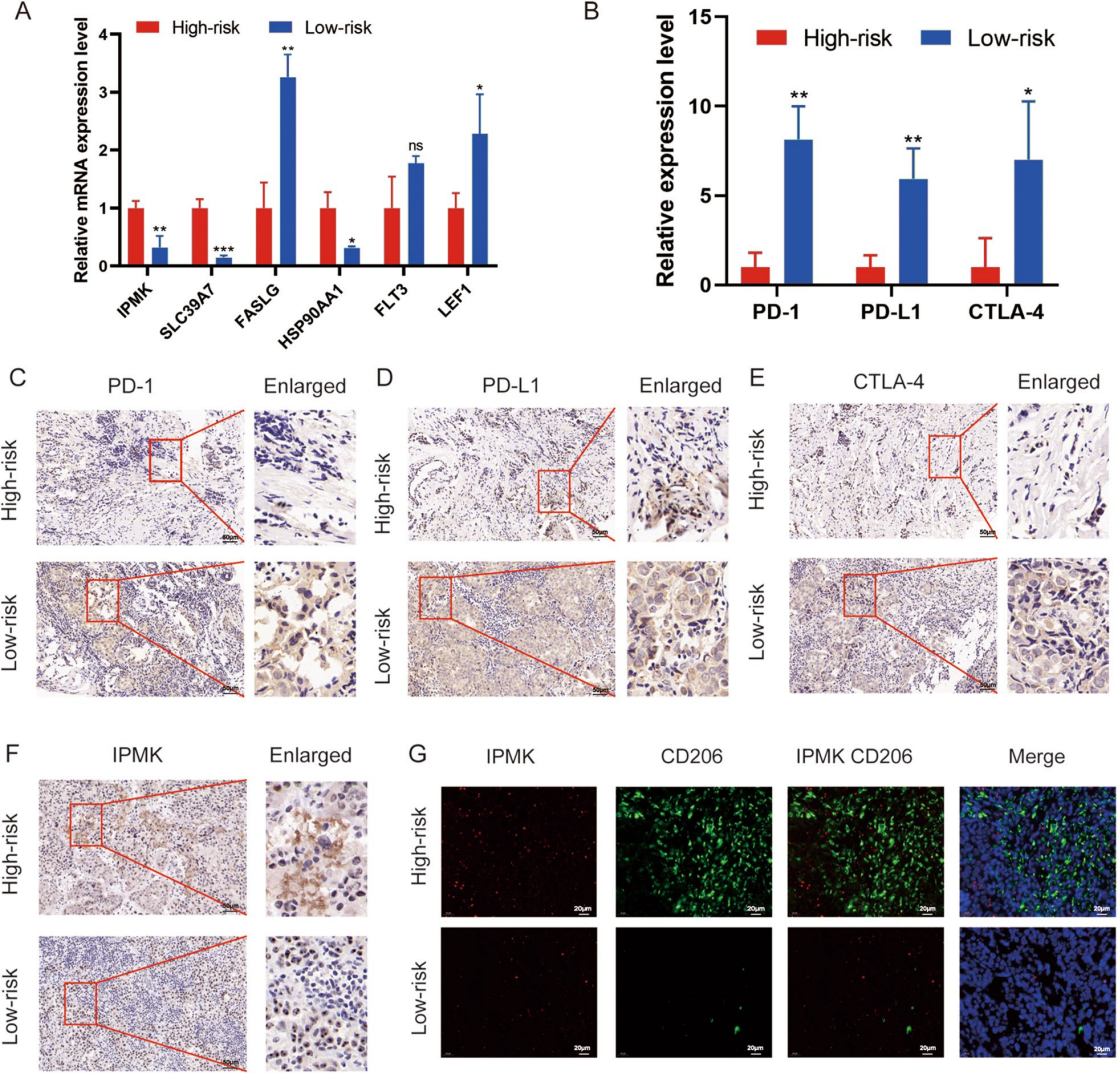
Validation of the NRG signature in an external clinical cohort. The expression of 6 selected NRGs (IPMK, SLC39A7, FASLG, HSP90AA1, FLT3, LEF1) (**A**) and immune checkpoints (PD-1, PD-L1, and CTLA-4) (**B**) in the two groups were detected using qRT-PCR. Immunohistochemistry was used to compare the expression of PD-1 (**C**), PD-L1 (**D**), CTLA-4 (**E**), and IPMK (**F**) in the two risk groups. **G** The co-expression of CD206 and IPMK was detected using immunofluorescence. ns, not significant; *P < 0.5; **P < 0.01; ***P < 0.001.

The external clinical cohort of 20 BC patients was used to validate the signature. The risk score of every BC patient was calculated based on the relative mRNA expression level of 6 selected NRGs (Fig. 11A). Based on the median risk score, the cohort was divided into high and low-risk groups. In addition, the expression levels of immune checkpoint PD-1, PD-L1, and CTLA-4 were higher in the low-risk group (Fig. 11B). Our cohort was consistent with those of the TCGA database. The IHC also proved the high expression of PD-1, PD-L1, and CTLA-4 in BC samples in the low-risk group (Fig. 11C–E). Also, IPMK was associated with poor prognosis in our signature and was overexpressed in the high-risk group (Fig. 11F). IF showed that expression of M2 macrophage marker CD206 and IPMK were both higher in the high-risk group compared with the low-risk group (Fig. 11G).

### Biological functions and immunomodulatory functions of the selected gene

**Fig. 12:**
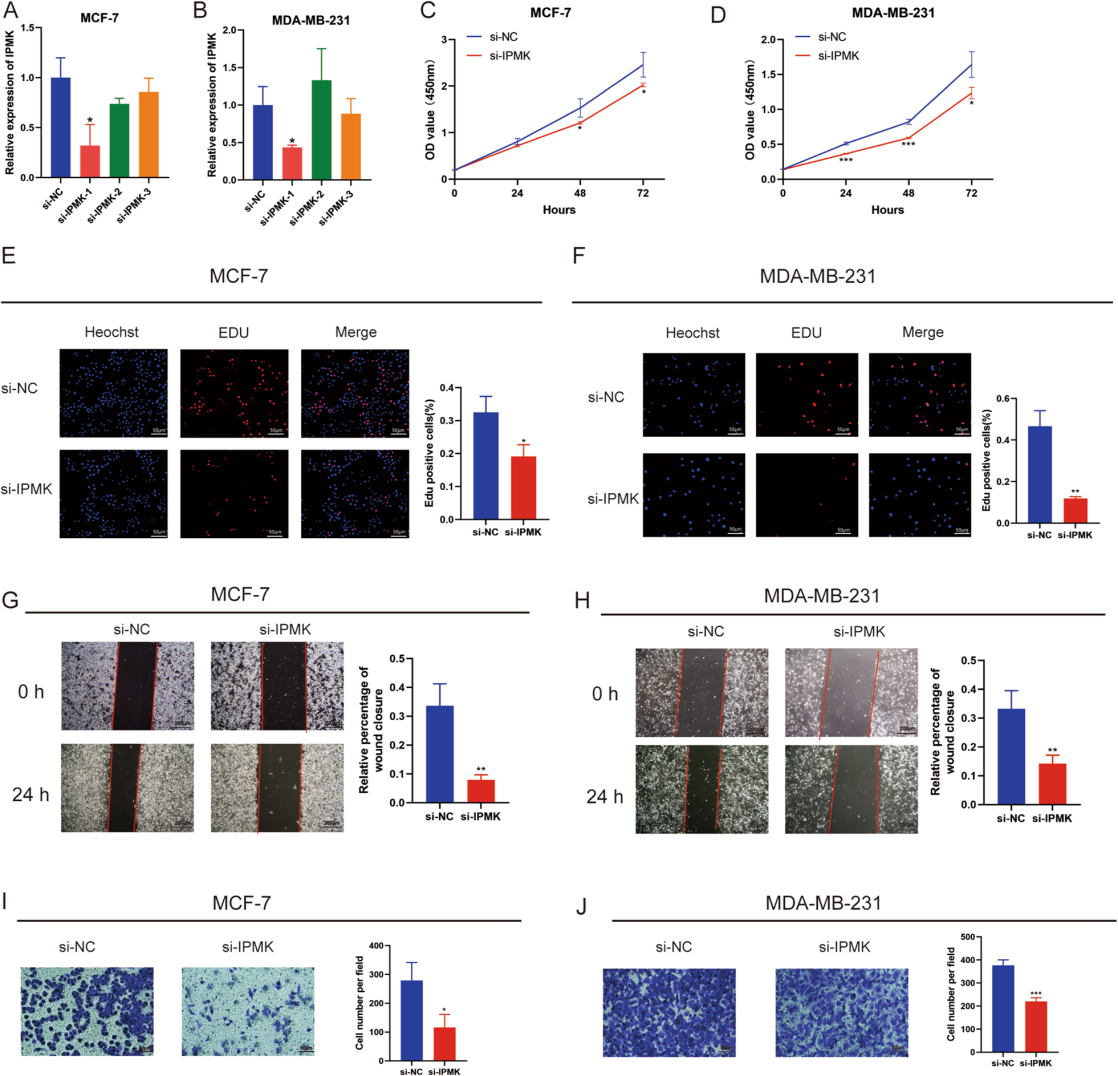
IPMK promoted the proliferation and migration of BC cells. The expression of IPMK in MCF-7 (**A**) and MDA-MB-231 (**B**) after transferring with si-IPMK was determined by qRT-PCR. The proliferation abilities of BC cells were detected by CCK-8 (**C**, **D**) and EdU assay (**E**, **F**). Cell migration was detected by wound healing (**G**, **H**) and Transwell assay (**I**, **J**). *P < 0.5; **P < 0.01; ***P < 0.001.

The silence efficiencies of siRNA in MCF-7 and MDA-MB-231 cell lines were evaluated by qRT-PCR (Fig. 12A, B). si-IPMK-1 was selected for the follow-up experiments. The results of CCK-8 assay and EdU assay proved that the silence of IPMK impaired the proliferation abilities of BC cells (Fig. 12C–F). Moreover, wound healing and transwell assay indicated that the migration of BC cells was suppressed after silencing of IPMK (Fig. 12G–J).

**Fig. 13:**
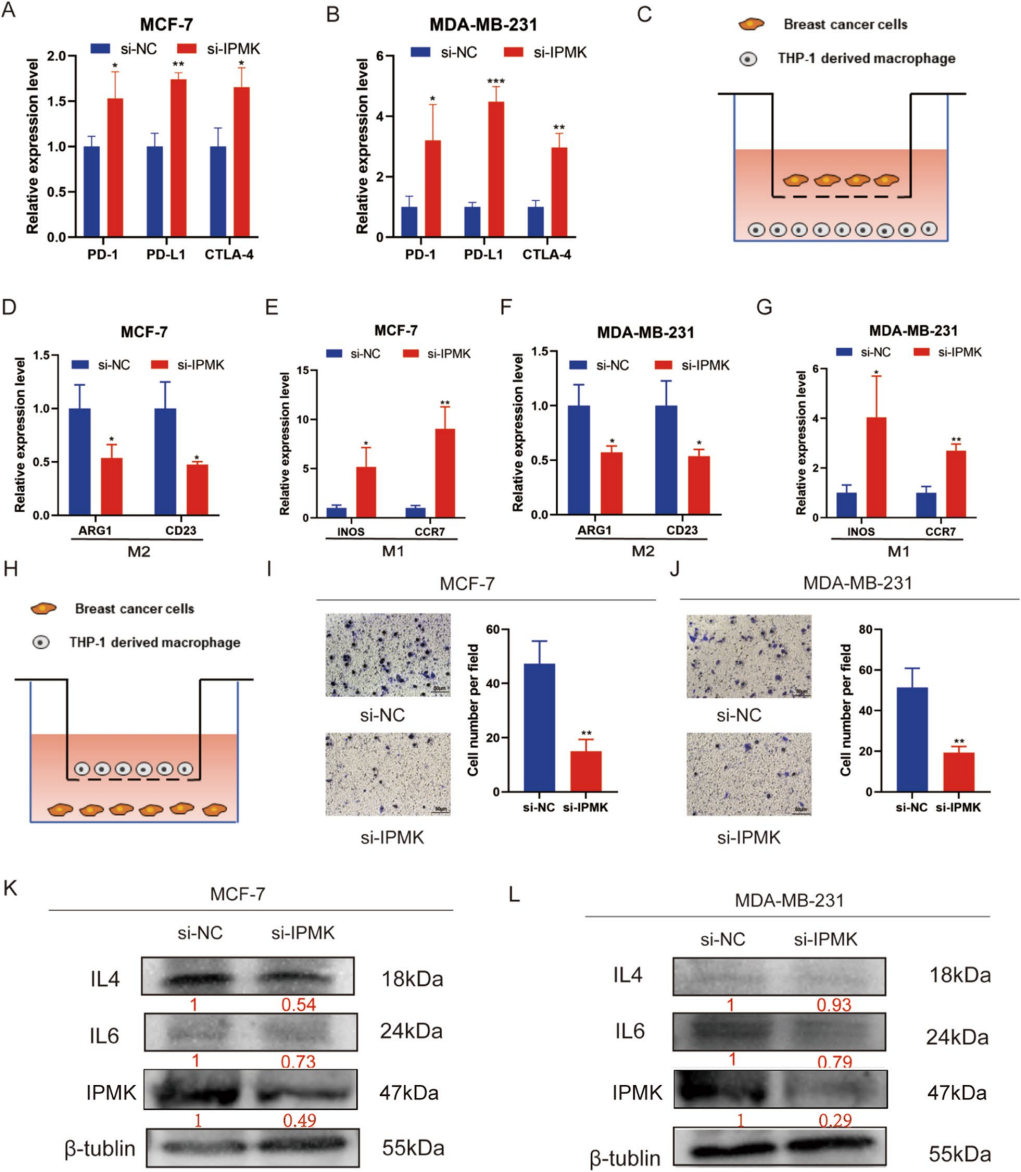
The silence of IPMK enhanced the expression of immune checkpoint and promoted infiltration and M2 polarization of macrophages. The relative expression levels of PD-1, PD-L1, and CTLA-4 in MCF-7 (**A**) and MDA-MB-231 cells (**B**) after silencing IPMK were compared with the si-NC groups. After co-culturing THP-1 generated M0 macrophages with BC cells (**C**) for 48 h with Transwell plates, the expressions of M2 biomarkers (ARG1 and CD23) and M1 biomarkers (INOS and CCR27) of macrophage (**D–G**) were detected by qRT-PCR. Then, the macrophages were seeded in the upper chamber of Transwell plates (**H**). The number of migrated macrophages after co-culture for 48 h with MCF-7 (**I**) and MDA-MB-231 (**J**) were calculated by Image J. The protein expressions of IL-4 and IL-6 of MCF-7 (**K**) and MDA-MB-231 cells (**L**) were detected using western blot analysis. *P < 0.5; **P < 0.01; ***P < 0.001.

Surprisingly, the expressions of immune checkpoint PD-1, PD-L1, and CTLA-4 were increased after silencing IPMK (Fig. 13A, B). Then we co-cultured BC cell lines and M0 macrophage generated from THP-1 cells (Fig. 13C). The silence of IPMK in BC cells reduced M2 macrophage biomarkers (ARG1 and CD23) expression and increased M1 macrophage biomarkers (INOS and CCR7) expression (Fig. 13D–G). In addition, we also evaluated the effect of IPMK on the macrophage recruitment function of BC cells (Fig. 13H). The silence of IPMK in BC cells inhibited macrophage migration (Fig. 13 I–J). Moreover, after the si-IPMK treatment, the IPMK protein expression level was significantly down-regulated. IPMK silence reduced the IL-4 and IL-6protein expression (Fig. 13K, L). The above results indicated that IPMK promoted the proliferation and migration of BC cells and was involved in tumor immunoregulation.

## Discussion

It is well known that BC is a type of cancer with high heterogeneity. The prognosis and responses to treatment vary greatly in patients with different molecular characteristics. Here, we established a NRG signature based on FASLG, IPMK, FLT3, SLC39A7, HSP90AA1, and LEF1, for the goal of predicting the prognosis, immunotherapy response, and chemosensitivity of BC patients. Firstly, our signature successfully distinguished patients with different prognosis, and its prediction efficiency were universal in different cohorts. Secondly, patients in the high-risk group had more aggressive PAM50 subtypes and lower levels of immune cell infiltration. Thirdly, the patients in the low-risk group were more likely to respond to immunotherapy and chemotherapy. Moreover, an external clinical cohort validated the results of the bioinformatic analysis. We also proved that IPMK was highly expressed in the high-risk groups, and could promote proliferation and migration of BC cells, as well as induce M1 to M2 macrophage polarization.

Resistance to apoptosis is considered to be an important hallmark of tumors [26]. Some cancer cells escape from necroptosis by decreasing the expression of key necroptosis mediators, including CYLD, RIPK3, and MLKL [27]. In addition, necroptosis creates an inflammatory milieu to regulate CD8+ T cell-mediated anti-tumor immunity [28]. Necroptosis is reported to play an important role in the progression of BC. In triple-negative breast cancer (TNBC), AQP1 overabundance inhibited RIPK1-mediated necroptosis and promoted progression and metastasis [29]. Zheng et al. reported that necroptosis-related miRNAs could predict the rate of metastasis of BC patients [30].

Here, in our study, the GO analysis proposed that differently expressed NRGs were markedly associated with the dominant top 5 terms of BP, MF, and CC. Especially, these NRGs showed an intensive relationship with many cell death models represented by necroptosis, as well as other proteins and cellular structures that influence tumor progression. The necroptosis-based pharmacological inhibition strategies have endowed huge potential in promoting human BC cell proliferation and metastasis [31]. The KEGG results showed that specific pathways, such as various cancer types and necroptosis, were enriched in NRGs. It is worth noting that TNF and NF-kB signaling pathways are enriched in differently expressed NRGs. TNFα/TNFR signaling pathway is the most well-known pathway for activating necroptosis. TNFα activates TNFR and recruits RIPK1, TNFR1-associated death domain protein, cellular inhibitors of apoptosis, and TNFR-associated factor 2 to form complex 1. When RIPK1 is deubiquitinated, RIPK1 combines with Fas-associated protein with a novel death domain and caspase 8 to form complex II and interacts with RIPK3 to induce necroptosis [32]. Wu et al. suggested that TNF-α enhanced the effect of chemotherapy by inducing RIP3-dependent necroptosis [33]. Tan et al. demonstrated that DRD2 inhibited the NF-kB signaling pathway and induced necroptosis in BC cells [34]. Thus, the TNF and NF-kB related with differently expressed NRGs are important orchestrators in shaping BC progression.

The function of 6 NRGs in our signature, including FASLG, IPMK, FLT3, SLC39A7, HSP90AA1, and LEF1, are studied in various cancer types, including BC. FASLA is a member of the tumor necrosis factor (TNF) family. FASLG-FAS interaction activated RIPK1 and produce necrosome and finally induced necroptosis in tumor cells [35]. Wang et al. demonstrated that the overexpression of lncRNA CASC7 increased the FASLG expression and promoted apoptosis of BC cells [36]. FLT3 is the most common mutation site in acute myeloid leukemia, and the mutation of FLT3 increased the expression level of RIPK1 and the sensitivities of necroptosis [37]. Recent studies indicated that zinc transporter SLC39A7 participated in regulating TNFR1-mediated necroptosis and activated endoplasmic reticulum stress in cells [38]. High expression of SLC39A7 was related to a worse prognosis in BC patients [39]. BC patients with high expression of HSP90AA1 in plasma had lower OS and a higher risk of metastasis [40]. LEF1 was a key regulator involved in TNFα/zVAD-induced necroptosis [41]. Vila et al. demonstrated that LEF1 inhibition in BC cells enhanced their response to docetaxel [42].

As the fact that necroptosis always results in a strong inflammatory response, we explored the correlation between risk score and the immune infiltration and response to immunotherapy and chemotherapy. Checkpoint blockades (anti-PD-1, anti-PD-L1, and CTLA-4) have reached remarkable success in many types of cancers [43]. In BC, anti-PD-1 (pembrolizumab) combined with chemotherapy is approved for the treatment of TNBC in the early stage in the United States. anti-PD-L1 (Atezolizumab) was also approved for the treatment of PD-L1+ metastatic BC in other countries. A lot of ongoing clinical trials were exploring the effect of checkpoint blockade [44]. The therapeutic effect of ICIs is associated with the comprehensive impacts of various factors, such as immune checkpoint expression, TIDE, TMB, and related gene expression characteristics [45]. In our research, we found that the low-risk group had higher infiltration of most immune cells, TMB, TIDE, and immune checkpoint expression. Simultaneously, patients in the low-risk group were more sensitive to immunotherapy.

IPMK is a type of inositol phosphate kinases involved in the production of IP4 [46]. IPMK promoted MLKL oligomerization and membrane recruitment to active MLKL-mediated necroptosis [47]. Sei et al. indicated that mutant IPMK was the risk factor for small intestinal carcinoids [48]. Liu et al. demonstrated that inhibition of IPMK mediated by miR-18a inhibited ovarian tumor growth [49]. Our research firstly proved that IPMK promoted the progression of BC. Kim et al. showed that IPMK promoted TLR-dependent inflammation by binding to TRAF6 [50]. Wang et al. demonstrated that inhibition of IPMK/TRAF6 decreased the activity of osteoclast [51]. Previous research mainly focused on the function of IPMK in the regulation of macrophage and immune response. The role of IPMK in tumor immunity has not been studied previously. Our results showed that down-regulated IPMK expression in BC cells reduced M2 polarized macrophages and the infiltration of macrophages. IL-4 and IL-6 secreted by cancer cells could promote the infiltration and M2 polarization of macrophages [52]. Moreover, Pasparakis et al. demonstrated that necroptosis was involved in cytokine production [53]. We found that the knockdown of IPMK reduced the expression of IL-4 and IL-6 in BC cells.

There are still some deficiencies in our research. Our study was mainly based on the public databases, including TCGA, GEO, and IMvigor 210. The number of patients included in our external clinical cohort is not enough. Large clinical trials are needed to verify the accuracy of the results. Although the correlation between IPMK and tumor immunity has been preliminarily proved in our study, further research is needed to explore how IPMK regulates the immune microenvironment of BC.

## Conclusion

In conclusion, we successfully established the NRG signature based on FASLG, IPMK, FLT3, SLC39A7, HSP90AA1, and LEF1. The signature was an independent prognostic predictor and had better prediction efficiency than other signatures. Moreover, the low-risk group had high immune cell infiltration levels, high TMB, and better response to immunotherapy and chemotherapy. IPMK was found to promote BC progression and regulate tumor immunity. Our signature could be used to evaluate BC prognosis and identify patients well responding to immunotherapy for precisely combating BC.

## Supplementary Information

**Additional file 1:** **Table S1.** Primer sequences for mRNAs

**Additional file 2:** **Table S2.** Results of univariate regression analysis of all 43 NRG genes.

**Additional file 3:** **Table S3.** Results of multivariate regression analysis of all 43 NRG genes.

**Additional file 4:** **Figure S1.** Enrichment analysis of differently expressed NRGs. (A) Heat map for differently expressed NRGs. (B) Visualization of top 5 enriched GO analyses in BP, MF, and CC. (C) Visualization of top 30 enriched KEGG pathways.

## Data Availability

All the datasets displayed in this study can be obtained in the online database. Further questions can be directed to the corresponding author.

## Abbreviations

EdU: 5-Ethynyl-20-Deoxyuridine
AUC: Area under the curve
BP: Biological process
BC: Breast cancer
CAF: Cancer associated fiblast
CCK-8: Cell counting kit-8
CC: Cellular component
cDNA: Complementary DNA
CR: Complete response
CAN: Copy number alteration
CTLA-4: Cytotoxic T-lymphocyte-associated antigen
DFS: Disease-free survival
ECL: Enhanced chemiluminescence
GEO: Gene expression omnibus
GDSC: Genomics of drugs sensitivity in cancer
IF: Immuinofluorescence
ICIs: Immune checkpoint inhibitors
IRRs: Infusion-related reactions
IPMK: Inositol polyphosphate multikinase
MLKL: Mixed lineage kinase domain-like
IHC: Mmunohistochemistry
MF: Molecular function
MDSC: Myeloid-derived suppressor cells
NRG: Necroptosis-related gene
OS: Overall survival
PR: Partial response
PMA: Phorbol-12-myristate-13-acetate
PVDF: Polyvinylidene difluoride
PD-L1: Programmed cell death 1 ligand 1
PD-1: Programmed cell death receptor 1
PFI: Progression-free interval
PD: Progressive disease
qRT-PCR: Quantitative real-time polymerase chain reaction
RIPK3: Receptor-interacting serine-threonine kinase 3
SDS-PAGE: Sodium dodecyl sulfate–polyacrylamide gel electrophoresis
SD: Stable disease
TCGA: The cancer genome atlas
TRIM28: Tripartite motif protein 28
TNBC: Triple-negative breast cancer
TMB: Tumor mutation burden
TNF: Tumor necrosis factor
TAM: Tumor-associated macropgage
