# Supplementary material for: Immunotherapy landscape analyses of necroptosis characteristics for breast cancer patients
Source: J Transl Med. 2022 Jul 21;20:328. doi: 10.1186/s12967-022-03535-z (PMC9306193; doi:10.1186/s12967-022-03535-z)
Supplement: Supplementary file 1 — Additional file 1: Table S1. Primer sequences for mRNAs [file 12967_2022_3535_MOESM1_ESM.docx]

| mRNAs | **Forward Primer** | **Reverse Primer** |
| --- | --- | --- |
| PD-1 | CCAGGATGGTTCTTAGACTCCC | TTTAGCACGAAGCTCTCCGAT |
| PD-L1 | GCTGCACTAATTGTCTATTGGGA | AATTCGCTTGTAGTCGGCACC |
| CTLA-4 | GCCCTGCACTCTCCTGTTTTT | GGTTGCCGCACAGACTTCA |
| IPMK | AGGACAACTGTCAGACACAGA | TGACGCGCATACATCTTGGAC |
| SLC39A7 | GGACACGCTCACAGTCATACA | CTCCTCGCCTCTTCTGAACC |
| FASL9 | TGCCTTGGTAGGATTGGGC | GCTGGTAGACTCTCGGAGTTC |
| HSP90AA1 | AGGAGGTTGAGACGTTCGC | AGAGTTCGATCTTGTTTGTTCGG |
| FLT3 | AGGGACAGTGTACGAAGCTG | GCTGTGCTTAAAGACCCAGAG |
| LEF1 | AGAACACCCCGATGACGGA | GGCATCATTATGTACCCGGAAT |
| ARG1 | GTGGAAACTTGCATGGACAAC | AATCCTGGCACATCGGGAATC |
| CD23 | GCGTGGGACTCAGATCGTG | GCTGTTTTAGACTCTGTGTGGTG |
| iNOS | TTCAGTATCACAACCTCAGCAAG | TGGACCTGCAAGTTAAAATCCC |
| CCR7 | TGAGGTCACGGACGATTACAT | GTAGGCCCACGAAACAAATGAT |

**Table S1.** Primer sequences for mRNAs
