# Supplementary material for: Immunotherapy landscape analyses of necroptosis characteristics for breast cancer patients
Source: J Transl Med. 2022 Jul 21;20:328. doi: 10.1186/s12967-022-03535-z (PMC9306193; doi:10.1186/s12967-022-03535-z)
Supplement: Supplementary file 2 — Additional file 2: Table S2. Results of univariate regression analysis of all 43 NRG genes. [file 12967_2022_3535_MOESM2_ESM.docx]

Table S2 Results of univariate regression analysis of all 43 NRG genes.

| id | HR | HR.95L | HR.95H | pvalue |
| --- | --- | --- | --- | --- |
| FASLG | 0.806557 | 0.654907 | 0.993324 | 0.043074 |
| IPMK | 1.092513 | 1.01916 | 1.171145 | 0.012589 |
| FLT3 | 0.886297 | 0.812349 | 0.966975 | 0.006618 |
| SLC39A7 | 1.005872 | 1.001063 | 1.010704 | 0.016642 |
| HSP90AA1 | 1.001113 | 1.000531 | 1.001695 | 0.000175 |
| LEF1 | 0.965124 | 0.934754 | 0.996481 | 0.02955 |
| FADD | 1.003589 | 0.982467 | 1.025166 | 0.741312 |
| FAS | 0.973397 | 0.929464 | 1.019407 | 0.252513 |
| MLKL | 0.95717 | 0.840732 | 1.089735 | 0.508322 |
| RIPK1 | 0.998859 | 0.953709 | 1.046146 | 0.961404 |
| RIPK3 | 0.949062 | 0.874771 | 1.029662 | 0.208711 |
| TLR3 | 0.892502 | 0.777904 | 1.023982 | 0.104809 |
| TNF | 0.971185 | 0.902454 | 1.04515 | 0.434947 |
| TSC1 | 1.028343 | 0.928954 | 1.138366 | 0.58994 |
| TRIM11 | 1.001282 | 0.932984 | 1.07458 | 0.971642 |
| CASP8 | 0.950609 | 0.857033 | 1.054401 | 0.338041 |
| ZBP1 | 0.917068 | 0.799154 | 1.05238 | 0.217616 |
| MAPK8 | 0.986269 | 0.910536 | 1.068301 | 0.734478 |
| ITPK1 | 0.981031 | 0.957904 | 1.004716 | 0.115629 |
| SIRT3 | 0.950048 | 0.891967 | 1.011912 | 0.111372 |
| MYC | 1.000869 | 0.9967 | 1.005055 | 0.683485 |
| TNFRSF1A | 0.993198 | 0.978581 | 1.008034 | 0.36694 |
| TNFRSF1B | 0.978332 | 0.959226 | 0.997819 | 0.029487 |
| TRAF2 | 1.001717 | 0.966813 | 1.037882 | 0.924464 |
| PANX1 | 1.043548 | 1.010975 | 1.07717 | 0.008424 |
| OTULIN | 1.00142 | 0.941219 | 1.065472 | 0.964214 |
| CYLD | 1.015063 | 0.967577 | 1.06488 | 0.5408 |
| AXL | 0.998461 | 0.971467 | 1.026206 | 0.912323 |
| ID1 | 0.991096 | 0.975335 | 1.007111 | 0.274143 |
| CDKN2A | 0.985661 | 0.965296 | 1.006455 | 0.175138 |
| HSPA4 | 1.009899 | 1.001231 | 1.018642 | 0.025114 |
| BCL2 | 0.993322 | 0.978879 | 1.007978 | 0.369931 |
| STUB1 | 1.004181 | 0.987364 | 1.021284 | 0.628263 |
| HAT1 | 1.013061 | 0.972971 | 1.054802 | 0.528765 |
| SIRT2 | 1.019159 | 0.982526 | 1.057158 | 0.309582 |
| SIRT1 | 1.003159 | 0.96127 | 1.046874 | 0.884761 |
| PLK1 | 1.022133 | 0.999829 | 1.044934 | 0.051801 |
| MPG | 0.985522 | 0.964972 | 1.006509 | 0.174949 |
| BACH2 | 0.726381 | 0.517994 | 1.018601 | 0.063864 |
| IDH2 | 1.001665 | 0.999812 | 1.003521 | 0.078224 |
| BNIP3 | 1.020802 | 1.004819 | 1.037039 | 0.010557 |
| CD40 | 0.957123 | 0.918804 | 0.99704 | 0.035538 |
| BCL2L11 | 1.010803 | 0.969987 | 1.053336 | 0.609401 |
