# Supplementary material for: Immunotherapy landscape analyses of necroptosis characteristics for breast cancer patients
Source: J Transl Med. 2022 Jul 21;20:328. doi: 10.1186/s12967-022-03535-z (PMC9306193; doi:10.1186/s12967-022-03535-z)
Supplement: Supplementary file 3 — Additional file 3: Table S3. Results of multivariate regression analysis of all 43 NRG genes. [file 12967_2022_3535_MOESM3_ESM.docx]

Table S3 Results of multivariate regression analysis of all 43 NRG genes.

| Id | coef | HR | HR.95L | HR.95H | pvalue |
| --- | --- | --- | --- | --- | --- |
| FASLG | -0.23338 | 0.791852 | 0.639835 | 0.979987 | 0.031885 |
| IPMK | 0.070337 | 1.07287 | 0.998184 | 1.153144 | 0.038059 |
| FLT3 | -0.08917 | 0.914688 | 0.844034 | 0.991256 | 0.0297 |
| SLC39A7 | 0.004625 | 1.004636 | 0.999965 | 1.009329 | 0.041737 |
| HSP90AA1 | 0.000878 | 1.000878 | 1.000233 | 1.001524 | 0.007623 |
| LEF1 | -0.02173 | 0.978504 | 0.948731 | 1.009211 | 0.016808 |
