# Supplementary figures and images for: Immunotherapy landscape analyses of necroptosis characteristics for breast cancer patients
Source: J Transl Med. 2022 Jul 21;20:328. doi: 10.1186/s12967-022-03535-z (PMC9306193; doi:10.1186/s12967-022-03535-z)

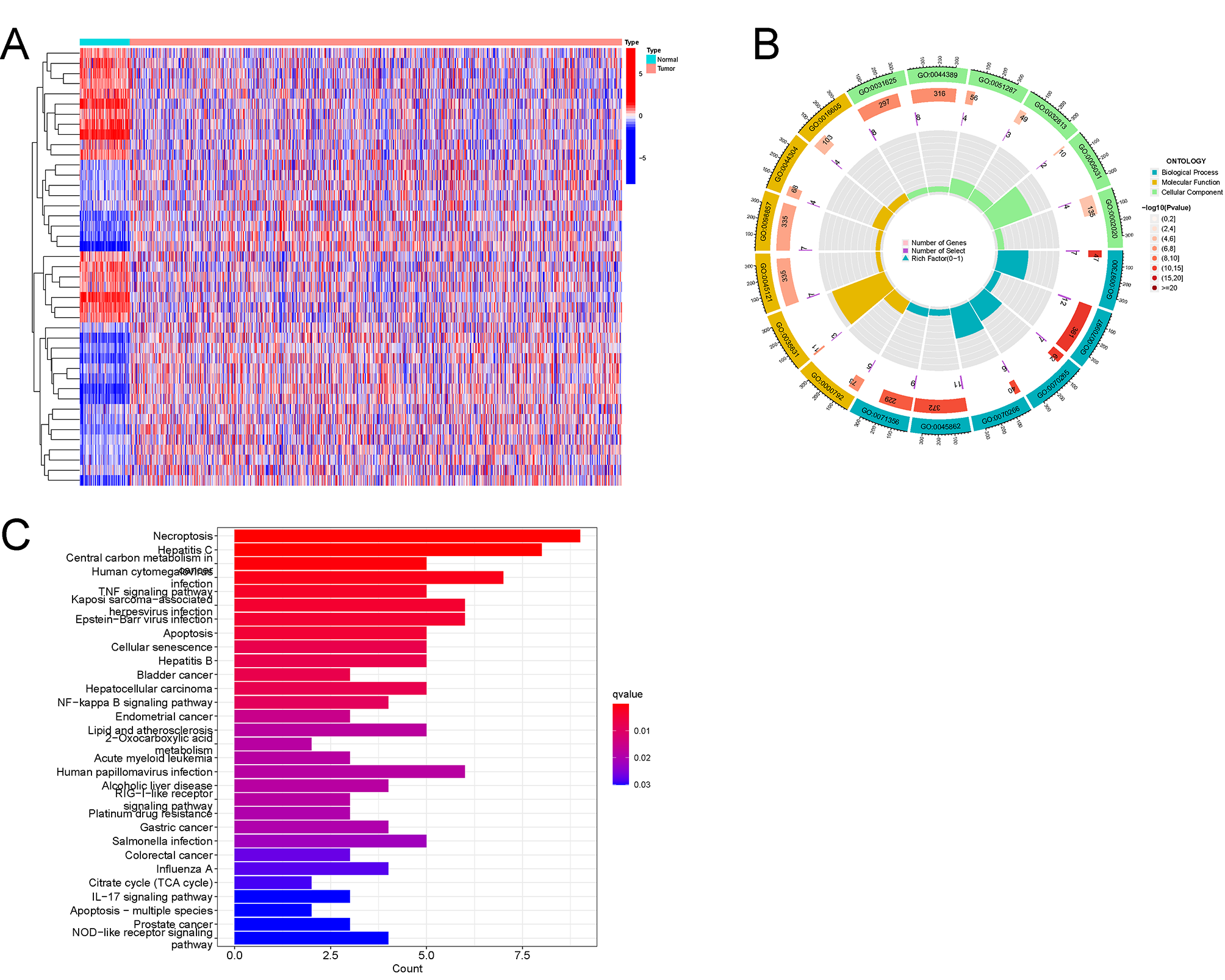

Supplement: Supplementary file 4 — Additional file 4: Figure S1. Enrichment analysis of differently expressed NRGs. (A) Heat map for differently expressed NRGs. (B) Visualization of top 5 enriched GO analyses in BP, MF, and CC. (C) Visualization of top 30 enriched KEGG pathways. [file 12967_2022_3535_MOESM4_ESM.tif]
